# Mechanofluorochromism of (D–π–)_2_A-type azine-based fluorescent dyes†

**DOI:** 10.1039/d2ra02431d

**Published:** 2022-05-10

**Authors:** Kosuke Takemura, Keiichi Imato, Yousuke Ooyama

**Affiliations:** Applied Chemistry Program, Graduate School of Advanced Science and Engineering, Hiroshima University 1-4-1 Kagamiyama Higashi-Hiroshima 739-8527 Japan yooyama@hiroshima-u.ac.jp

## Abstract

Bathochromic or hypsochromic shift-type mechanofluorochromism (b-MFC or h-MFC) was found for (D–π–)_2_A-type azine-based fluorescent dyes OUY-2, OUK-2, and OUJ-2 possessing intramolecular charge-transfer (ICT) characteristics from two (diphenylamino)carbazole–thiophene units as D (electron-donating group)–π (π-conjugated bridge) moieties to a pyridine, pyrazine, or triazine ring as A (electron-withdrawing group): grinding of the recrystallized dyes induced red or blue shifts of the fluorescent colors, that is, bathochromic or hypsochromic shifts of the fluorescence maximum wavelengths (λ^fl-solid^_max_). The degrees of MFC evaluated by the absolute value of differences (Δ*λ*^fl-solid^_max_) in λ^fl-solid^_max_ before and after grinding of the recrystallized dyes increased in the order of OUY-2 (+7 nm) < OUK-2 (−17 nm) < OUJ-2 (+45 nm), so that OUJ-2 exhibits obvious b-MFC, but OUK-2 exhibits h-MFC. X-ray powder diffraction (XRD) and differential scanning calorimetry (DSC) demonstrated that the recrystallized dyes were in the crystalline state but the ground dyes were in the amorphous state. When the ground solids were heated above their crystallization temperatures (*T*_c_), the colors and fluorescent colors recovered to the original ones before grinding or converted to other ones, that is, heating the ground solids in the amorphous state induced the recrystallization to recover the original microcrystals or to form other microcrystals due to polymorph transformation. However, (D–π–)_2_Ph-type fluorescent dye OTK-2 having a phenyl group as a substitute for the azine rings exhibited non-obvious MFC. Molecular orbital (MO) calculations indicated that the values of the dipole moments (*μ*_g_) in the ground state were 4.0 debye, 1.4 debye, 3.2 debye, and 2.9 debye for OTK-2, OUY-2, OUK-2, and OUJ-2, respectively. Consequently, on the basis of experimental results and MO calculations, we have demonstrated that the MFC of the (D–π–)_2_A-type azine-based fluorescent dyes is attributed to reversible switching between the crystalline state of the recrystallized dyes and the amorphous state of the ground dyes with changes in the intermolecular dipole–dipole and π–π interactions before and after grinding. Moreover, this work reveals that (D–π–)_2_A fluorescent dyes possessing dipole moments of *ca.* 3 debye as well as moderate or intense ICT characteristics make it possible to activate the MFC.

## Abstract 2

Bathochromic or hypsochromic shift-type mechanofluorochromism (b-MFC of h-MFC) was found for (D–π–)_2_A-type azine-based fluorescent dyes: grinding of the recrystallized dyes induced bathochromic or hypsochromic shifts of the fluorescence bands.

## Graphical abstract

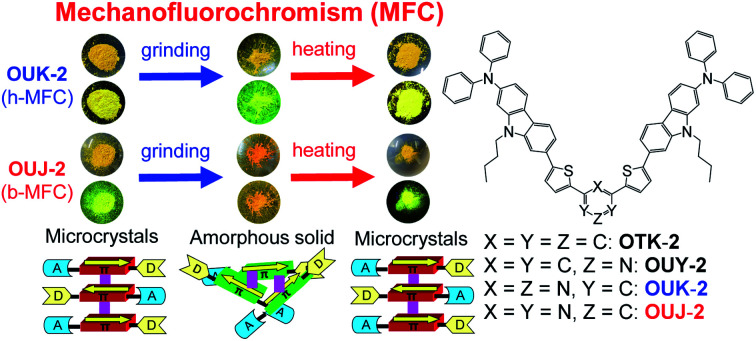

## Introduction

**Fig. 1:**
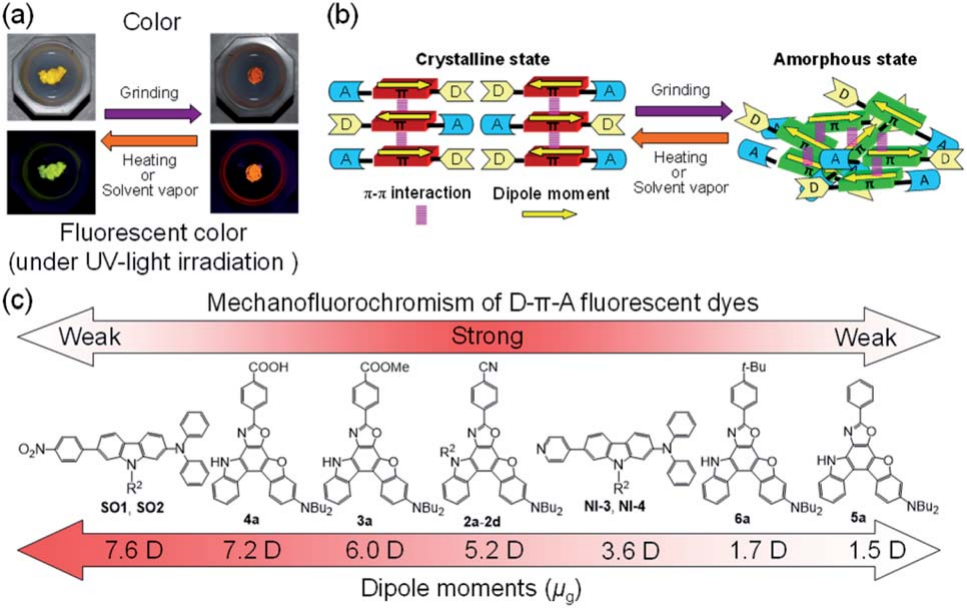
(a) Photographs of powder of heteropolycyclic D–π–A fluorescent dye under room light (top) and under UV-light irradiation (down) before and after grinding and after heating the ground solid. (b) Mechanisms of MFC observed in heteropolycyclic D–π–A fluorescent dyes. (c) Correlation between dipole moments and MFC characteristics of D–π–A fluorescent dyes.

Mechanofluorochromism (MFC) is a phenomenon that induces changes in fluorescent colors as well as colors of organic or metal complex fluorescent dyes in the solid state by external mechanical stimuli, being accompanied by reversion to the original colors and fluorescent colors by heating or exposure to solvent vapor.^1–7^ In many cases, MFC is based on reversible changes in the electronic structures of dye molecules and/or intermolecular interactions between the dye molecules induced by changes in the chemical structures (cleavage and reconstruction of chemical bonds and twisting and distortion of the structures) or molecular orientation and arrangement of dye molecules, which are altered by solid-state transformation (crystal-to-crystal and crystal-to-amorphous phase transitions) by grinding and heating or exposure to solvent vapor, resulting in changes in the photoabsorption and fluorescence properties. In particular, the intermolecular MFC (inter-MFC) based on reversible changes in molecular orientation and arrangement of dye molecules is superior in durability to the intramolecular MFC (intra-MFC) based on reversible changes in the chemical structures of dye molecules by cleavage and reconstruction of chemical bonds during the grinding–heating process, and thus, the inter-MFC is expected to be applicable to rewritable photoimaging and electroluminescence devices.^8–70^ Among various types of fluorescent dyes possessing the mechanofluorochromic properties, donor–π–acceptor (D–π–A)-type fluorescent dyes composed of an electron-donating moiety (D) and an electron-withdrawing moiety (A) connected by a π-conjugated bridge exhibit intense photoabsorption and fluorescence emission properties based on the intramolecular charge transfer (ICT) excitation from the D moiety to the A moiety.^11–33^ Thus, the dipole moments as well as photoabsorption (*i.e.*, color) and fluorescence emission (*i.e.*, fluorescent color) wavelengths of D–π–A-type fluorescent dyes are tunable by not only the electron-donating ability of D and the electron-withdrawing ability of A but also the electronic characteristics of the π-conjugated bridge. The D–π–A-type fluorescent dyes are well known to undergo significant fluorescence quenching (*i.e.*, ACQ: aggregation-caused quenching) and bathochromic shifts of the photoabsoprtion and fluorescence maximum wavelengths (*λ*^abs^_max_ and *λ*^fl^_max_) from the solution to the solid state due to the delocalization of excitons or excimers by the formation of intermolecular π–π interactions between the dye molecules in the solid state, which can be controlled by changing the steric hindrance of substituents on the D and A moieties and/or π-conjugated bridge.^11–16,71–73^ Therefore, the D–π–A-type fluorescent dyes occasionally show the intra-MFC based on the twisting and distortion between the π-conjugated bridge and D or A moiety or the inter-MFC based on changes in the intermolecular dipole–dipole and π–π interactions between the dye molecules. Most D–π–A-type mechanofluorochromic dyes developed so far exhibit bathochromic shift-type MFC (b-MFC): grinding of the recrystallized dyes induces red shifts of the fluorescent colors, that is, bathochromic shifts of *λ*^fl^_max_. Indeed, in our previous study, we have reported that newly developed heteropolycyclic D–π–A-type fluorescent dyes possessing moderate dipole moments (*μ*_g_ = *ca.* 5–6 debye) in the ground state show distinguished inter-b-MFC (Fig. 1a).^6,11–13^ Our work revealed that the inter-b-MFC of the D–π–A-type fluorescent dyes is attributed to reversible switching between the crystalline and amorphous states with changes in intermolecular dipole–dipole and π–π interactions between the dye molecules (Fig. 1b). Meanwhile, it was found that D–π–A-type fluorescent dyes possessing large dipole moments (*μ*_g_ = *ca.* 8 debye) show weak MFC due to the strong dipole–dipole interactions between the dye molecules, changes in which may be inhibited in the crystal-to-amorphous phase transition.^14^ Moreover, it was suggested that negligible MFC observed in D–π–A-type fluorescent dyes possessing small dipole moments (*μ*_g_ = *ca.* 1–2 debye) is ascribable to small changes in the dipole–dipole interactions between the crystalline and amorphous states.^15^ Consequently, we proposed that the most important point for developing MFC dyes is to design D–π–A-type fluorescent dye molecules possessing moderate dipole moments (*ca.* 5 debye), which are precisely controlled by tuning the electron-donating ability of D, electron-withdrawing ability of A, steric size of substituents, and D–π–A system (Fig. 1c).^6^ In addition, for the relationship between *μ*_g_ and MFC, Zhang *et al.* have reported that tetraphenylethylene (TPE) derivatives possessing large dipole moments are closely packed in the amorphous phase due to their intense ICT characteristics and the strong dipole–dipole interactions and the ICT characteristics are enhanced by grinding due to the increased molecular planarity, resulting in pronounced b-MFC.^34^ In contrast, TPE derivatives possessing small dipole moments show non-obvious MFC due to their weak ICT characteristics and dipole–dipole interactions. Recently, Thomas III *et al.* have investigated the MFC of D–π–A-type phenylene ethynylenes (PEs).^17–20^ They demonstrated that PEs having a strong D group exhibit b-MFC due to the formation of planarized/aggregated PEs by grinding, whereas PEs having a relatively weak D group exhibit hypsochromic shift-type MFC (h-MFC) due to the disruption of PE aggregates by grinding. Indeed, Misra *et al.* have reviewed D–A-type fluorescent dyes exhibiting h-MFC, such as triphenylamine-, TPE-, and benzothiadiazole-based derivatives.^7^ Meanwhile, Yamanoi *et al.* have reported that disilane-bridged D–A–D triad composed of phenothiazine and thienopyrazine exhibits b-MFC based on changes in conformational relaxation due to the flexibility of the Si–Si bond and phenothiazine groups between the crystalline and amorphous states.^74^ Therefore, in order to achieve pronounced and desired MFC that can control fluorescence properties by external mechanical stimuli, it is necessary to provide a direction in the molecular design toward creating mechanofluorochromic dyes to meet the above requirements.

**Fig. 2:**
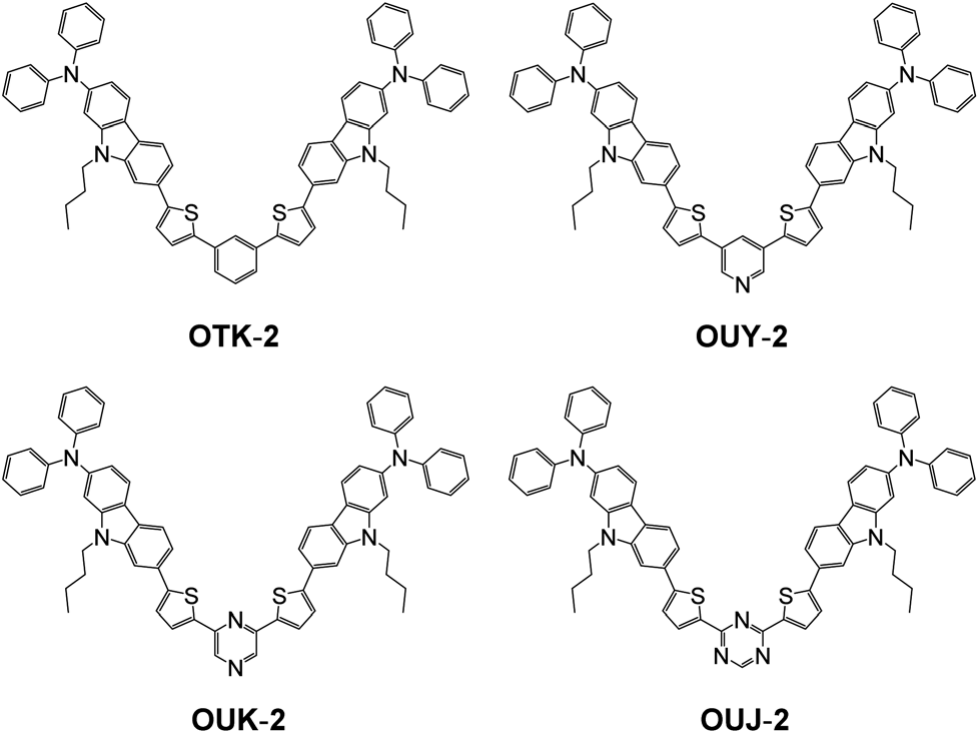
Chemical structures of (D–π–)_2_Ph fluorescent dye OTK-2 and (D–π–)_2_A-type azine-based fluorescent dyes OUY-2, OUK-2, and OUJ-2.

Thus, in this work, in order to further gain insight into the development of D–π–A-type mechnofluorochromic dyes, we have designed and synthesized (D–π–)_2_A-type azine-based fluorescent dyes OUY-2, OUK-2, and OUJ-2 with two (diphenylamino)carbazole–thiophene units as the D–π moiety and a pyridine, pyrazine, or triazine ring as the A group^75^ and a (D–π–)_2_Ph fluorescent dye OTK-2 having a phenyl (Ph) group as a substitute for the azine rings (Fig. 2). The (D–π–)_2_A-type fluorescent dyes are expected to show obvious MFC, that is, high fluorescent color contrast before and after grinding, because they generally exhibit high molar extinction coefficient and fluorescence quantum yield due to the intense ICT characteristics, compared with D–π–A-type fluorescent dyes. The presence or absence of MFC has been investigated from the photoabsorption, fluorescence excitation, and fluorescence spectral measurements, time-resolved fluorescence spectroscopy, X-ray powder diffraction (XRD), differential scanning calorimetry (DSC), and density measurements of the solids before and after grinding and after heating the ground solids. The MFC of (D–π–)_2_A-type azine-based fluorescent dyes were estimated from the viewpoint of the degree of *μ*_g_ based on molecular orbital (MO) calculations and changes in the intermolecular dipole–dipole and π–π interactions before and after grinding. Herein we reveal the effect of *μ*_g_ of (D–π–)_2_A-type fluorescent dyes on the MFC and propose the direction in molecular design toward creating D–π–A-type mechnofluorochromic dyes.

## Results and discussion

The (D–π–)_2_A-type azine-based fluorescent dyes OUY-2, OUK-2, and OUJ-2 were synthesized according to a stepwise synthetic protocol that has been reported elsewhere.^75^ In a similar way to OUY-2, OUK-2, and OUJ-2, the (D–π–)_2_Ph fluorescent dye OTK-2 was prepared by Stille coupling of a stannyl thiophene derivative having a (diphenylamino)carbazole moiety with 1,3-diiodobenzene (see Scheme S1 for the synthesis, ESI†).

**Fig. 3:**
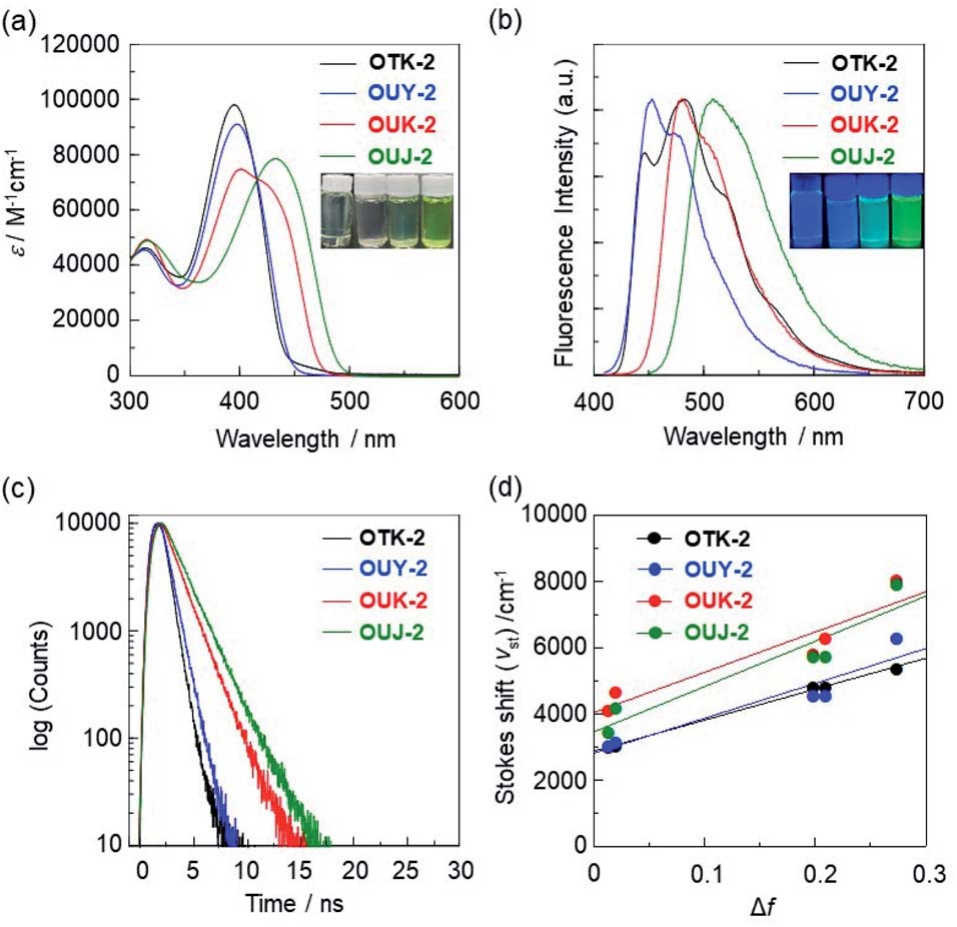
(a) Photoabsorption and (b) fluorescence (*λ*^ex^ = *λ*^abs^_max_) spectra and (c) fluorescence decay profiles of OTK-2, OUY-2, OUK-2, and OUJ-2 in toluene. (d) Lippert–Mataga plots of the Stokes shifts (*ν*_st_) *versus* orientation polarizabilities of solvents (Δ*f*; 0.0132 for toluene, 0.0205 for 1,4-dioxane, 0.199 for ethyl acetate, 0.2096 for THF, and 0.274 for DMF). The slopes (*m*_sl_) became steep in the order of OTK-2 (9339 cm^−1^) < OUY-2 (10 500 cm^−1^) < OUK-2 (12 200 cm^−1^) < OUJ-2 (13 700 cm^−1^). The correlation coefficient (*R*^2^) values for the calibration curves are 0.99 for OTK-2, 0.90 for OUY-2, 0.88 for OUK-2, and 0.89 for OUJ-2 and indicate good linearity. Insets in (a) and (b): color and fluorescence images of OTK-2, OUY-2, OUK-2, and OUJ-2 (from left) in toluene.

In our previous work, we demonstrated that OUY-2, OUK-2, and OUJ-2 exhibited an ICT-based photoabsorption band (*λ*^abs^_max_), which appeared in a longer wavelength region in the order of OUY-2 < OUK-2 < OUJ-2 in solution, in agreement with the increase in the electron-withdrawing ability of the azine rings in the order of pyridyl group < pyrazyl group < triazyl group (Fig. 3a and Tables 1, S2†).^75^ Interestingly, the photoabsorption spectra of OUK-2 show a shoulder band in the long-wavelength region close to the *λ*^abs^_max_ of OUJ-2. In toluene, OUY-2 and OUK-2 exhibited a vibronically-structured fluorescence band, while a broad fluorescence band was observed in OUJ-2 (Fig. 3b). Moreover, the three (D–π–)_2_A-type fluorescent dyes showed positive solvatofluorochromism (p-SFC): their photoabsorption spectra were nearly independent of solvent polarity, while their fluorescence spectra were dependent on solvent polarity, leading to bathochromic shifts of the fluorescence bands with increasing solvent polarity from toluene to DMF (Fig. S2†). OUK-2 and OUJ-2 exhibited significant SFC, that is, significant decreases in the fluorescence quantum yields (*Φ*_fl_) in polar solvents such as DMF compared with OUY-2 (Table S1†). The relationships between the *λ*^fl^_max_ and solvent polarity parameters were investigated on the basis of the Lippert–Mataga equation.^76–78^ The Lippert–Mataga plots of the Stokes shifts (*ν*_st_) *versus* the orientation polarizabilities (Δ*f*) of solvents revealed that the change in dipole moment, Δ*μ* = *μ*_e_ − *μ*_g_, between the ground (*μ*_g_) and excited (*μ*_e_) states increased in the order of OUY-2 (22 debye) < OUK-2 (25 debye) < OUJ-2 (26 debye), which corresponds to the increase in the electron-withdrawing ability of the azine rings (pyridyl group < pyrazyl group < triazyl group (Fig. 3d)). This result indicates that for OUY-2, OUK-2, and OUJ-2, the solvent-dependent bathochromic shifts in the fluorescence bands are mainly attributed to the dipole–dipole interactions between the fluorescent dye molecules and solvent molecules.^79–84^ For the case of OTK-2, meanwhile, the photoabsorption properties (*λ*^abs^_max_ and the molar extinction coefficient (*ε*_max_)) were nearly independent of solvent polarity as with the case of OUY-2, OUK-2, and OUJ-2, while the fluorescence properties (*λ*^fl^_max_ and *Φ*_fl_) were slightly affected by solvent polarity, resulting in weak SFC (Fig. S2 and Table S1†). The ICT-based photoabsorption and fluorescence bands of OTK-2 appeared in a shorter wavelength region than those of OUY-2, OUK-2, and OUJ-2 (Fig. 3), and OTK-2 exhibited a vibronic-structured fluorescence band in solvents except for DMF, due to the lack of electron-withdrawing ability of the phenyl group. In fact, the Lippert–Mataga plots demonstrated that the Δ*μ* value (21 D) of OTK-2 was much smaller than those of OUY-2, OUK-2, and OUJ-2 (Fig. 3d). The time-resolved fluorescence spectroscopy demonstrated that the fluorescence lifetimes (*τ*_fl_) in toluene increased in the order of OTK-2 (0.62 ns) < OUY-2 (0.82 ns) < OUK-2 (1.60 ns) < OUJ-2 (1.92 ns) (Fig. 3c and Table 1). It was also found that for the four fluorescent dyes, the *τ*_fl_ values decreased with increasing solvent polarity from toluene to DMF (0.62–1.07 ns for OTK-2, 0.82–2.33 ns for OUY-2, 1.60–2.14 ns for OUK-2 and 1.92–2.93 ns for OUJ-2, respectively, Table S1†). Consequently, the fact indicates that OUK-2 and OUJ-2 show salient SFC attributed to the moderate and intense ICT characteristics, respectively, due to the moderate and strong electron-withdrawing ability of the pyrazine and triazine rings, respectively. As shown in insets of Fig. 3a and b, in toluene, the colors are nearly-colorless for OTK-2 and OUY-2 and greenish-yellow for OUK-2 and OUJ-2, and the fluorescent colors are blue for OUY-2 and OTK-2, light blue for OUK-2, and green for OUJ-2.

**Table 1:** Optical data of OTK-2, OUY-2, OUK-2, and OUJ-2 in toluene

Dye	*λ* ^abs^_max_/nm (*ε*_max_/M^−1^ cm^−1^)	*λ* ^fl^_max_/nm (*Φ*_fl_)^a^	SS^b^/cm^−1^	*τ*_fl_ c/ns
OTK-2	395 (98 000)	447, 483 (0.36)	2945	0.62
OUY-2	398 (91 100)	453, 473 (0.38)	3051	0.82
OUK-2	401 (74 800)	480 (0.48)	4104	1.60
OUJ-2	433 (78 500)	509 (0.81)	3448	1.92

a Fluorescence quantum yields (*Φ*_fl_) were determined by using a calibrated integrating sphere system (*λ*^ex^ = *λ*^abs^_max_).

b Stokes shift.

c Fluorescence lifetime.

**Fig. 4:**
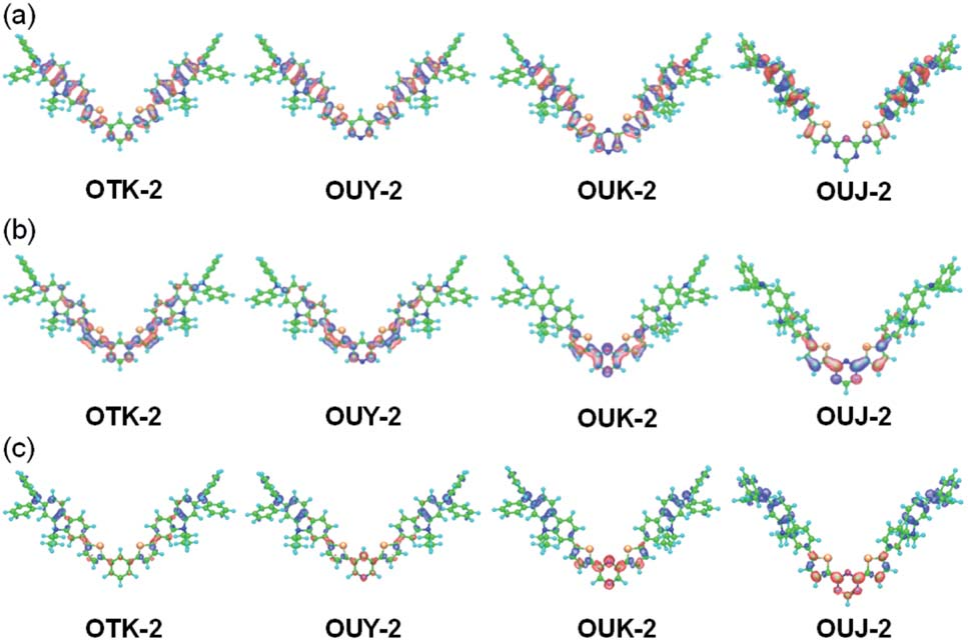
(a) HOMO and (b) LUMO of OTK-2, OUY-2, OUK-2, and OUJ-2 derived from MO calculations (PM5, INDO/S method). The red and blue lobes denote the positive and negative signs of the coefficients of the molecular orbitals. The size of each lobe is proportional to the MO coefficient. (c) Calculated electron density changes accompanying the first electronic excitation of OTK-2, OUY-2, OUK-2, and OUJ-2. The blue and red lobes signify decrease and increase in electron density accompanying the electronic transition, respectively. Their areas indicate the magnitude of the electron density changes.

The semi-empirical molecular orbital (MO) calculations (PM5, INDO/S method) showed that the calculated dihedral angles between the thiophene ring and carbazole skeleton were 44.1° for OTK-2, 44.5° for OUY-2, 43.6° for OUK-2, and 43.3° for OUJ-2, which were similar to each other (Fig. 4). On the other hand, the calculated dihedral angles between the thiophene ring and benzene or azine ring were 42.7° for OTK-2, 41.7° for OUY-2, 23.3° for OUK-2, and 1.3° for OUJ-2. The fact is not surprising since for OTK-2 and OUY-2, the two rings were expected to greatly twist due to the steric hindrance between the hydrogen atoms of the benzene or pyridine ring and hydrogen and sulfur atoms of the thiophene ring, while for OUK-2, the lack of steric hindrance between the hydrogen atom of the pyrazine ring and sulfur atom of the thiophene ring would lead to the relatively small dihedral angle. On the other hand, there is no such steric hindrance for OUJ-2, which results in the high coplanarity of the triazine ring and thiophene ring. Moreover, the MO calculations revealed that for OUY-2, OUK-2, and OUJ-2, the highest occupied molecular orbitals (HOMO) were mostly localized on the two (diphenylamino)carbazole moieties containing the thiophene ring, while the lowest unoccupied molecular orbitals (LUMO) were mostly localized on the thienylpyridine moiety for OUY-2, the thienylpyrazine moiety for OUK-2, and the thienyltriazine moiety for OUJ-2 (Fig. 4a and b). On the other hand, for OTK-2, both the HOMO and LUMO were mostly localized on the two (diphenylamino)carbazole moieties containing the thienylbenzene moiety. The changes in the calculated electron density accompanied by the first electron excitation for the four dyes are shown in Fig. 4c, which demonstrates the ICT characteristics from the two (diphenylamino)carbazole moieties (D–π moieties) to each azine ring (A unit) upon photoexcitation. It is worth noting here that the change in the calculated electron density for OUJ-2 is larger than those for OUY-2 and OUK-2, indicating that OUJ-2 possesses the intense ICT characteristics. The HOMO energy levels of the four dyes were similar to each other (−6.88 eV, −6.91 eV, −6.85 eV, and −7.00 eV for OTK-2, OUY-2, OUK-2, and OUJ-2, respectively), while the LUMO energy levels were lowered in the order of OTK-2 (−0.08 eV) > OUY-2 (−0.13 eV) > OUK-2 (−0.40 eV) > OUJ-2 (−0.58 eV). As the result, the HOMO–LUMO band gaps decreased in the order of OTK-2 (6.80 eV) > OUY-2 (6.78 eV) > OUK-2 (6.45 eV) > OUJ-2 (6.42 eV). Consequently, the MO calculations demonstrated that the bathochromic shifts of the ICT-based photoabsorption bands in the order of OTK-2 < OUY-2 < OUK-2 < OUJ-2 were attributed to stabilization of the LUMO energy levels due to the increase in the electron-withdrawing ability of the azine rings in the order of pyridyl group < pyrazyl group < triazyl group, as well as the increase in coplanarity of the azine or benzene ring and thiophene ring. Moreover, it was found that the values of the dipole moments (*μ*_g_) in the ground state were 4.0 debye, 1.4 debye, 3.2 debye, and 2.9 debye for OTK-2, OUY-2, OUK-2, and OUJ-2, respectively, and keep the fact in mind because we will discuss their MFC from the viewpoint of the intermolecular dipole–dipole interactions later.

The MFC characteristics of the (D–π–)_2_A and (D–π–)_2_Ph-type fluorescent dyes were investigated from the photoabsorption, fluorescence excitation, and fluorescence spectral measurements, time-resolved fluorescence spectroscopy, X-ray powder diffraction (XRD), differential scanning calorimetry (DSC), and density measurements of the solids before and after grinding and after heating the ground solids.

**Fig. 5:**
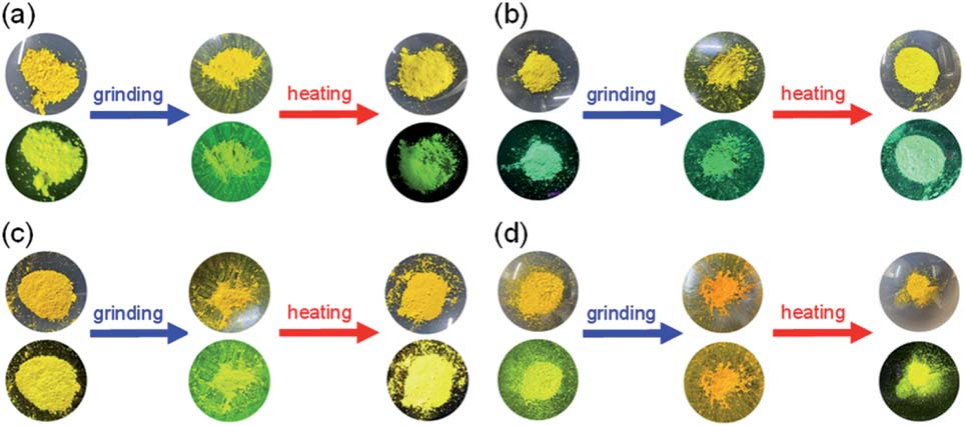
Photographs of powder of (a) OTK-2, (b) OUY-2, (c) OUK-2, and (d) OUJ-2 under room light (top) and under UV-light irradiation (down) before and after grinding of as-prepared microcrystalline dyes and after heating the ground solids.

**Fig. 6:**
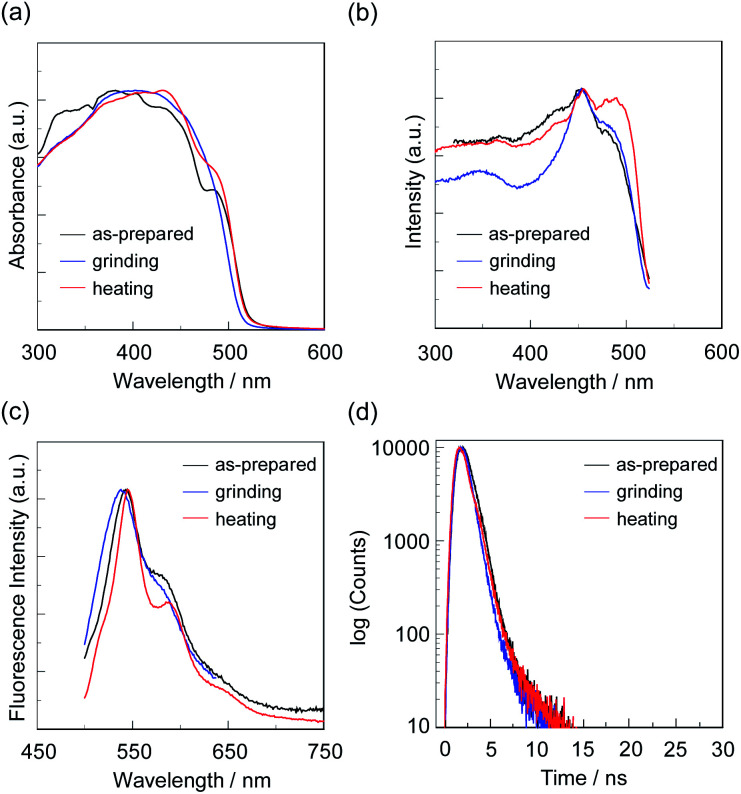
(a) Solid-state UV-vis diffuse reflection–absorption, (b) fluorescence excitation at λ^fl-solid^_max_, and (c) fluorescence spectra (*λ*^ex^ = *λ*^ex-solid^_max_) and (d) fluorescence decay profiles of OTK-2 before (as-prepared) and after grinding and after heating the ground solid at 200 °C.

**Fig. 7:**
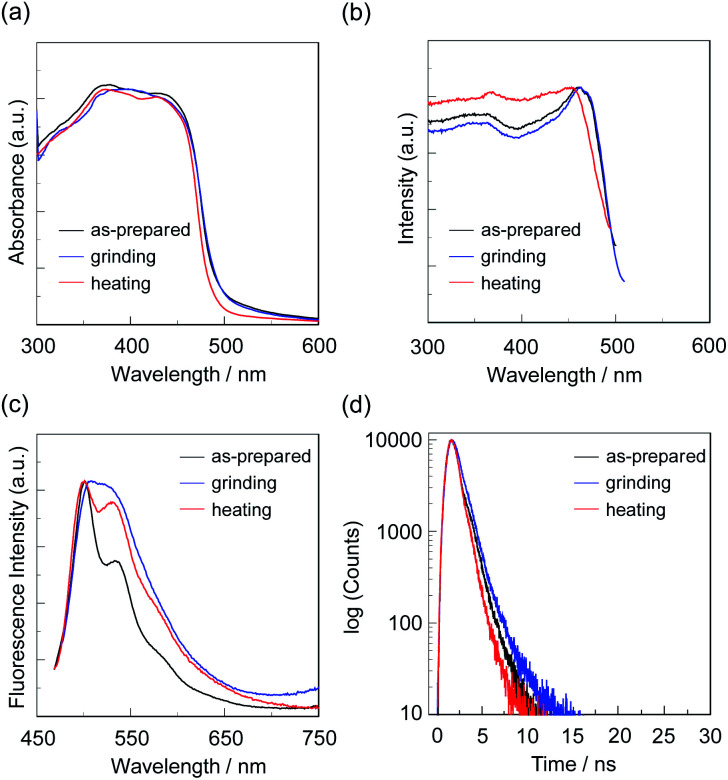
(a) Solid-state UV-vis diffuse reflection–absorption, (b) fluorescence excitation at λ^fl-solid^_max_, and (c) fluorescence spectra (*λ*^ex^ = *λ*^ex-solid^_max_) and (d) fluorescence decay profiles of OUY-2 before (as-prepared) and after grinding and after heating the ground solid at 200 °C.

**Fig. 8:**
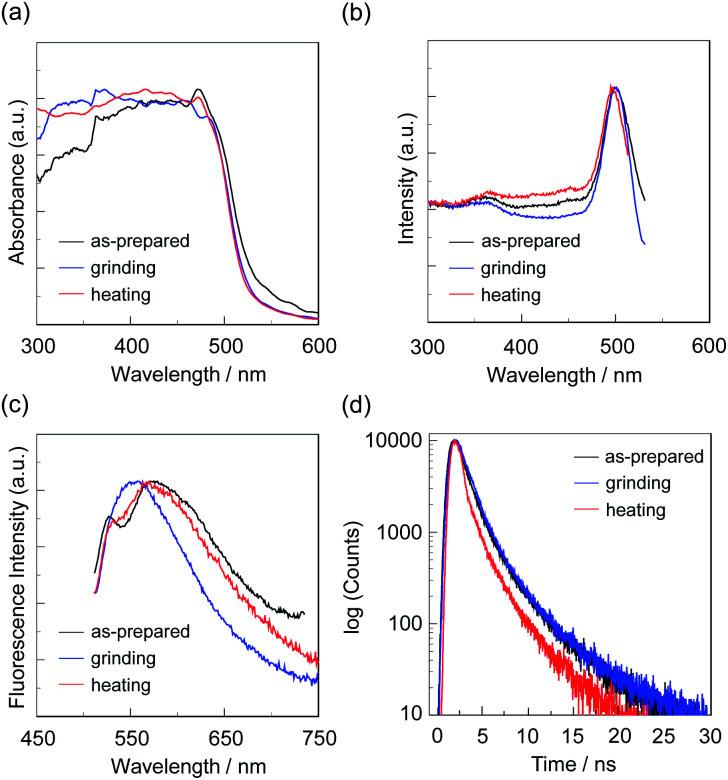
(a) Solid-state UV-vis diffuse reflection–absorption, (b) fluorescence excitation at λ^fl-solid^_max_, and (c) fluorescence spectra (*λ*^ex^ = *λ*^ex-solid^_max_) and (d) fluorescence decay profiles of OUK-2 before (as-prepared) and after grinding and after heating the ground solid at 180 °C.

**Fig. 9:**
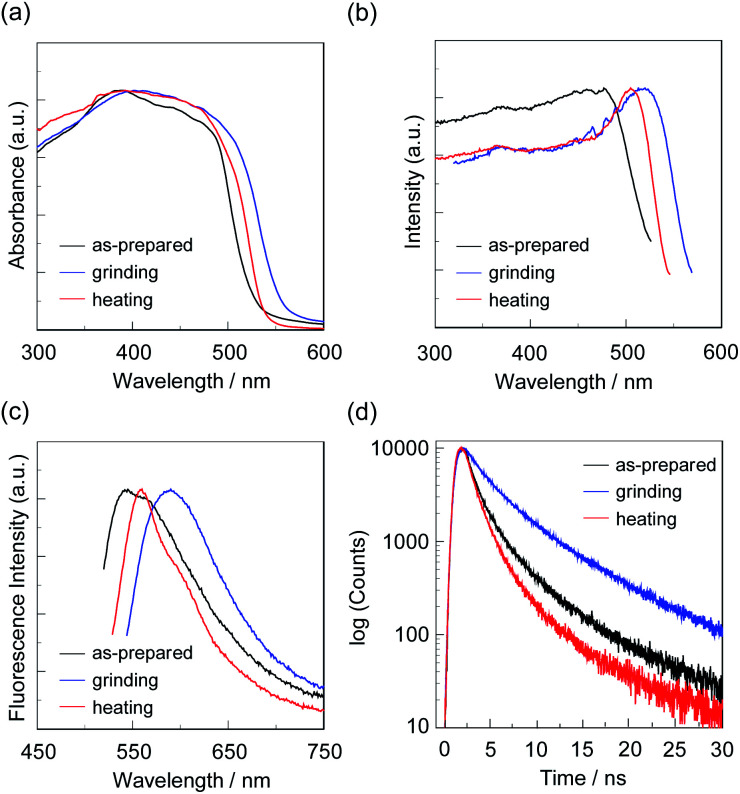
(a) Solid-state UV-vis diffuse reflection–absorption, (b) fluorescence excitation at λ^fl-solid^_max_, and (c) fluorescence spectra (*λ*^ex^ = *λ*^ex-solid^_max_) and (d) fluorescence decay profiles of OUJ-2 before (as-prepared) and after grinding and after heating the ground solid at 200 °C.

**Fig. 10:**
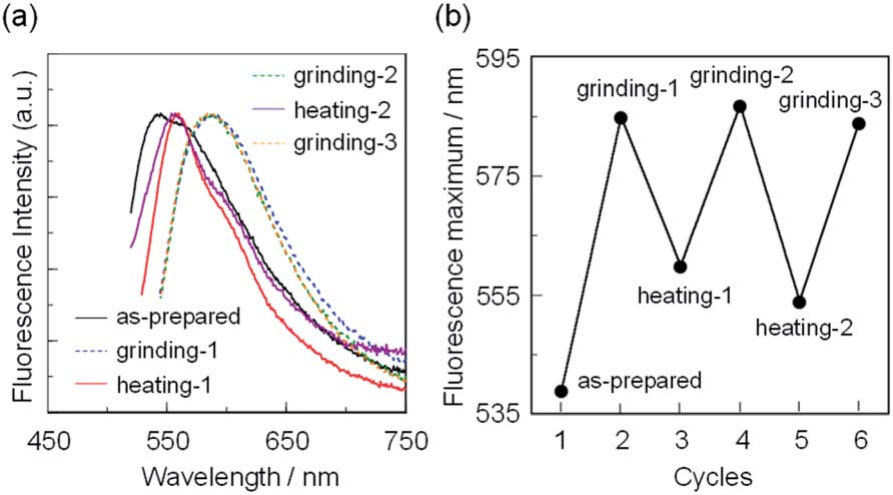
(a) Solid-state fluorescence spectra (*λ*^ex^ = *λ*^ex-solid^_max_) of OUJ-2 before (as-prepared) and after grinding and heating process. (b) Reversible switching of fluorescence maximum wavelength of OUJ-2 during the grinding-heating process.

First, microcrystals of OTK-2, OUY-2, OUK-2, and OUJ-2 were grown by slow evaporation of the dichloromethane/*n*-hexane solutions at room temperature for several days, and then, the as-recrystallized dyes were heated at 200 °C to yield the as-prepared microcrystalline dyes for the investigation of MFC. As the result, the colors of the as-prepared dyes were yellowish orange for OTK-2 and OUY-2 and yellow for OUK-2 and OUJ-2, and the fluorescent colors were greenish yellow for OTK-2 and OUJ-2, light green for OUY-2, and yellow for OUK-2 (Fig. 5). By grinding the as-prepared dyes at a stress of *ca.* 50 N cm^−2^ in a mortar with a pestle, the color and fluorescent color of the as-prepared OUJ-2 transformed into orange. The fluorescent color of the as-prepared OUK-2 transformed into greenish yellow by grinding, while there was no change in the color. On the other hand, the as-prepared OTK-2 and OUY-2 did not show obvious changes in the colors and fluorescent colors by grinding. The photoabsorption and fluorescence excitation maximum wavelengths (*λ*^abs-solid^_max_ and *λ*^ex-solid^_max_) of the as-prepared dyes appeared at 480 nm and 484 nm for OTK-2, 463 nm and 461 nm for OUY-2, 472 nm and 501 nm for OUK-2, and 482 nm and 477 nm for OUJ-2, respectively, which showed significant bathochromic shifts by 85 nm and 89 nm, 65 nm and 63 nm, 71 nm and 100 nm, and 49 nm and 44 nm, respectively, compared with those in toluene (Fig. 6–9a and b). The corresponding fluorescence maximum wavelengths (λ^fl-solid^_max_) of the as-prepared dyes appeared at 543 nm for OTK-2, 501 nm for OUY-2, 575 nm for OUK-2, and 545 nm for OUJ-2, which exhibited bathochromic shifts by 96 nm for OTK-2, 48 nm for OUY-2, 95 nm for OUK-2, and 36 nm for OUJ-2, compared with those in toluene (Fig. 6–9c). Interestingly, as with the case of the toluene solutions, the as-prepared dyes OTK-2, OUY-2, and OUK-2 exhibited a vibronically-structured fluorescence band, whereas broad fluorescence spectra with shoulder at 527 nm and 562 nm were observed in OUK-2 and OUJ-2, respectively. The fluorescence quantum yield (*Φ*_fl-solid_) values of the as-prepared dyes were 0.15 for OTK-2, 0.08 for OUY-2, 0.04 for OUK-2, and 0.12 for OUJ-2 (Table 2), which were significantly lower than those in toluene (Table 1). The bathochromic shifts of *λ*^abs^_max_ and *λ*^fl^_max_ and lowering of *Φ*_fl_ by changing from the solution to the solid state would be attributed to the formation of intermolecular π–π interactions between the fluorophores in the solid state and consequent delocalization of excitons or excimers,^71–73^ while unfortunately, we could not obtain single crystals of OTK-2, OUY-2, OUK-2, and OUJ-2 with sufficient size to make the X-ray structural analysis possible. The as-prepared OTK-2 did not exhibit any appreciable changes in the *λ*^abs-solid^_max_, *λ*^ex-solid^_max_, and λ^fl-solid^_max_ by grinding (Fig. 6a–c). For OUY-2 and OUK-2, the λ^fl-solid^_max_ were red-shifted and blue-shifted, respectively, while the *λ*^abs-solid^_max_ and *λ*^ex-solid^_max_ changed little by grinding (Fig. 7a–c and 8a–c). Thus, this result revealed that OUY-2 exhibits non-obvious b-MFC, but OUK-2 exhibits h-MFC. For OTK-2, OUY-2, and OUK-2, the vibronic structures in the fluorescence bands disappeared after grinding. Compared with OUY-2, on the other hand, OUJ-2 exhibited significant bathochromic shifts of the *λ*^abs-solid^_max_, *λ*^ex-solid^_max_, and λ^fl-solid^_max_ by grinding, that is, pronounced b-MFC (Fig. 9a–c). The degrees of MFC, which were evaluated by the absolute value of differences (Δ*λ*^abs-solid^_max_, Δ*λ*^ex-solid^_max_, and Δ*λ*^fl-solid^_max_) in *λ*^abs-solid^_max_, *λ*^ex-solid^_max_, and λ^fl-solid^_max_ before and after grinding of the as-prepared dyes, increased in the order of OUY-2 (n.d., n.d., −5 nm) < OUK-2 (0 nm, +2 nm, +7 nm) ≪ OUK-2 (+8 nm, 0 nm, −17 nm) < OUJ-2 (+42 nm, +43 nm, +45 nm), in agreement with the order of increase in the electron-withdrawing ability of the azine rings in the order of pyridyl group < pyrazyl group < triazyl group (Table 2). On the other hand, it is worth mentioning here that the Δ*λ*^fl-solid^_max_ between the shoulder fluorescence band of the as-prepared dyes and the λ^fl-solid^_max_ after grinding are +31 nm for OUK-2 and +28 nm for OUJ-2, in which this can be regarded as the b-MFC of OUK-2 as well as OUJ-2. The *Φ*_fl-solid_ values increased slightly by grinding from 0.15, 0.08, 0.04, and 0.12 to 0.16, 0.10, 0.06, and 0.17 for OTK-2, OUY-2, OUK-2, and OUJ-2, respectively (Table 2). Moreover, the time-resolved fluorescence spectroscopy revealed that the fluorescence lifetime (*τ*_fl-solid_) value of the as-prepared OTK-2 became shorter from 0.93 ns before grinding to 0.73 ns after grinding, whereas the *τ*_fl-solid_ values of OUY-2, OUK-2, and OUJ-2 became longer from 1.02 ns, 2.04 ns, and 2.14 ns before grinding to 1.05 ns, 2.16 ns, and 5.29 ns after grinding, respectively (Fig. 6–9d and Table 2). In particular, the *τ*_fl-solid_ value of OUJ-2 after grinding were 2.5 times longer than that before grinding. When the ground solids were heated at 180–200 °C (above the recrystallization temperatures (*T*_c_), as described later) or exposed to solvent vapor such as THF for several minutes (for OUJ-2, Fig. S4 and S5†), the photoabsorption, fluorescence excitation, and fluorescence bands of OTK-2 and OUK-2 as well as the colors and fluorescent colors recovered to the original ones before grinding, whereas the intensity of the vibronic structure in the fluorescence band of OUY-2 and the *λ*^abs-solid^_max_, *λ*^ex-solid^_max_, and λ^fl-solid^_max_ of OUJ-2 were different from the original ones before grinding. Besides, the fluorescence spectra of OUJ-2, which showed the pronounced b-MFC, after grinding and heating the ground solid were repeatedly measured several times (Fig. 10a). The fluorescence spectra showed a change in the λ^fl-solid^_max_ from 590 nm after grinding to 560 nm after heating. Indeed, the grinding-heating cycles indicated that OUJ-2 exhibited good reversibility of the λ^fl-solid^_max_ (Fig. 10b). Meanwhile, with regard to the chemical stability of the dyes by grinding and heating, the ^1^H NMR spectra of the samples after grinding and heating demonstrated that all the four fluorescent dyes were not decomposed by grinding and heating (for OUJ-2, Fig. S3†).

**Table 2:** Optical data of OTK-2, OUY-2, OUK-2, and OUJ-2 in the solid state

Dye	*λ* ^abs-solid^_max_/nm	Δ*λ*^abs-solid^_max_/nm	*λ* ^ex-solid^_max_/nm	Δ*λ*^ex-solid^_max_/nm	λ^fl-solid^_max_/nm (*Φ*_fl-solid_)^a^	Δ*λ*^fl-solid^_max_/nm	*τ*_fl-solid_ b/ns
OTK-2 (as-prepared)	480^shoulder^		484^shoulder^		543 (0.15)		0.93
		—c		0^d^		−5^d^	
OTK-2 (grinding)	—c		484^shoulder^		538 (0.16)		0.73
		—c		+4^e^		+7^e^	
OTK-2 (heating)	480^shoulder^		488		545 (0.19)		0.84
							
OUY-2 (as-prepared)	463^shoulder^		461		501 (0.08)		1.02
		0^d^		+2^d^		+7^d^	
OUY-2 (grinding)	463^shoulder^		463		508 (0.10)		1.05
		−3^e^		−10^e^		−7^e^	
OUY-2 (heating)	460^shoulder^		453		501 (0.12)		0.81
							
OUK-2 (as-prepared)	472		501		527^shoulder^, 575 (0.04)		2.04
		+8^d^		0^d^		+31, −17^d^	
OUK-2 (grinding)	480^shoulder^		501		558 (0.06)		2.16
		−8^e^		−6^e^		−31, +12^e^	
OUK-2 (heating)	472		495		527, 570 (0.04)		2.14
							
OUJ-2 (as-prepared)	482^shoulder^		477		545, 562^shoulder^ (0.12)		2.14
		+42^d^		+43^d^		+45, +28^d^	
OUJ-2 (grinding)	524^shoulder^		520		590 (0.17)		5.29
		−14^e^		−15^e^		−30^e^	
OUJ-2 (heating)	510^shoulder^		505		560 (0.13)		1.98

a Fluorescence quantum yields (*Φ*_fl-solid_) were determined by using a calibrated integrating sphere system (*λ*^ex^ = *λ*^ex-solid^_max_).

b Fluorescence lifetime.

c Due to broadened band.

d *λ* ^abs, ex, or fl-solid^_max_ (grinding) − *λ*^abs, ex, or fl-solid^_max_ (as-prepared).

e *λ* ^abs, ex, or fl-solid^_max_ (heating) − *λ*^abs, ex, or fl-solid^_max_ (grinding).

**Fig. 11:**
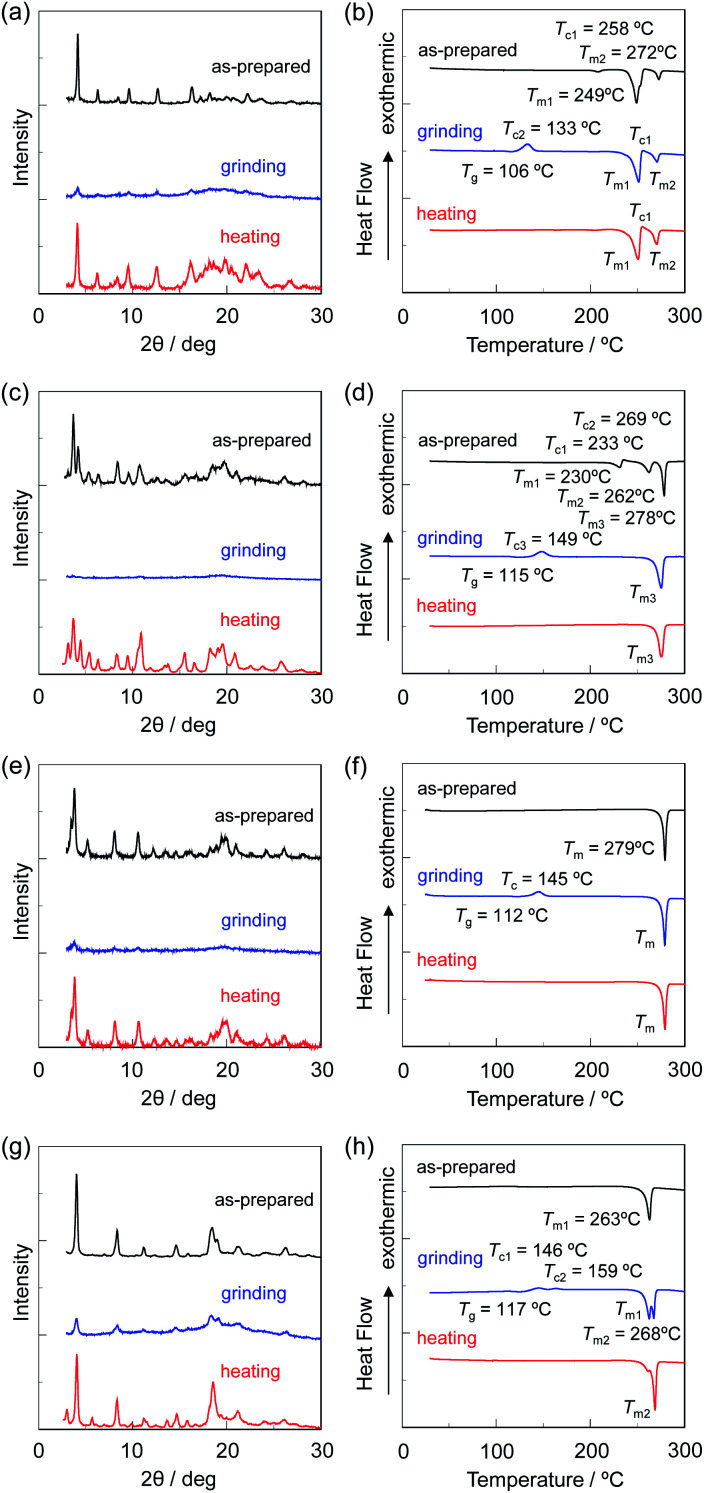
XRD patterns of (a) OTK-2, (c) OUY-2, (e) OUK-2, and (g) OUJ-2 and DSC curves (heating process from 25 °C to 300 °C with scan rate of 10 °C min^−1^) of (b) OTK-2, (d) OUY-2, (f) OUK-2, and (h) OUJ-2 before (as-prepared) and after grinding and after heating the ground solid.

The XRD measurements with the as-prepared dyes OTK-2, OUY-2, OUK-2, and OUJ-2 exhibited diffraction peaks ascribable to well-defined microcrystalline structures (Fig. 11a, c, e and g). The peaks disappeared almost completely after grinding, indicating that the crystal lattices were significantly disrupted. The diffraction peaks of OTK-2 and OUK-2 after heating the ground solids were similar to those before grinding, that is, the as-prepared dyes, indicating recovery of the microcrystalline structures by heating. On the other hand, the XRD patterns of OUY-2 and OUJ-2 after heating the ground solids showed diffraction peaks at around 2*θ* = 3° for OUY-2 and 2*θ* = 3°, 6°, and 12° for OUJ-2, which were not observed in those of the as-prepared dyes. Furthermore, the DSC analysis for the four fluorescent dyes indicated that the as-prepared dyes OUK-2 and OUJ-2 showed only one sharp endothermic peak associated with melting (*T*_m_) at 279 °C and 263 °C, respectively, but the DSC traces of the as-prepared dyes OTK-2 and OUY-2 were typical of polymorphic mixtures (Fig. 11b, d, f and h). The as-prepared dye OTK-2 showed a *T*_m1_ at 249 °C and then recrystallization (*T*_c_) at 258 °C, which in turn melted at 272 °C (*T*_m2_). Interestingly, OUY-2 has three crystal forms. The DSC traces of the as-prepared dye OUY-2 showed a *T*_m1_ at 230 °C, *T*_c1_ at 233 °C, *T*_m2_ at 262 °C, *T*_c2_ at 269 °C, and *T*_m3_ at 278 °C. The MFC for crystal polymorphism of OUY-2 will be reported elsewhere. The DSC traces of the ground solids of the four dyes were typical of amorphous solids: the ground solids underwent an endothermic glass transition (*T*_g_) and then an exothermic *T*_c_ before *T*_m_. It is worth noting here that by heating the ground solids in the amorphous state, OUY-2 did not recover the original microcrystalline structure but form a more stable microcrystalline structure showing *T*_m3_ at 278 °C, and OUJ-2 formed another microcrystalline structure showing *T*_m2_ at 268 °C, which was not observed in the DSC trace of the as-prepared microcrystal. After the ground solids of the four dyes were heated at 180–200 °C above the *T*_c_, the DSC traces showed only one *T*_m_ for OUY-2, OUK-2, and OUJ-2 but one *T*_c_ and two *T*_m_ for OTK-2 due to the crystal polymorphism. Consequently, the XRD and DSC studies revealed that heating the ground solids in the amorphous state induced the recrystallization to recover the original microcrystals or to form the other microcrystals due to the polymorph transformation. Moreover, it was found that as with the case of D–π–A-type fluorescent dyes,^11–15^ the MFC of the (D–π–)_2_A-type azine-based fluorescent dyes was not just a matter of events originating from a reversible change between crystalline and amorphous states by grinding and heating, because OUY-2 as well as OTK-2 exhibited non-obvious MFC. More interestingly, the densities of the solids measured by the Archimedian method were found to increase by grinding from 1.40, 1.26, 1.25, and 1.13 g cm^−3^ to 1.45, 1.37, 1.39, and 1.31 g cm^−3^ for OTK-2, OUY-2, OUK-2, and OUJ-2, respectively, indicating that the dye molecules in the amorphous state after grinding were more densely packed.

On the basis of the above experimental results and MO calculations, we discuss the mechanism of MFC observed in the (D–π–)_2_A fluorescent dyes below. In the crystalline state of the D–π–A and (D–π–)_2_A fluorescent dyes, the long range π–π interactions between adjacent molecular planes cause the stacking of the dye molecules so as to maximize the intermolecular dipole–dipole interactions by arranging the dipole moments in anti-parallel orientation, leading to the bathochromic shifts of *λ*^abs^_max_ and *λ*^fl^_max_ by changing from the solution to the crystalline state. Moreover, the lowering of *Φ*_fl_ from the solution to the crystalline state is attributed to the delocalization of excitons due to the formation of the long-range intermolecular π–π interactions in the crystalline state, leading to a non-radiative decay route for the excited states. By grinding the as-prepared microcrystalline dyes, on the other hand, the dye molecules would move closer to each other so as to maximize the dipole–dipole interactions (so as to arrange the adjacent dipole moments in head-to-tail orientation) as well as the intermolecular π–π interactions, as evidenced by the increased densities of the dyes in the amorphous state. The dipole–dipole interaction energy will be enhanced in dyes with greater dipole moments. In fact, the *μ*_g_ values of OUK-2 and OUJ-2 were 3.2 debye and 2.9 debye, respectively, which were greater than 1.4 debye for OUY-2, reflecting that OUK-2 and OUJ-2 have the π-conjugated planes with the D–π moieties and strongly electron-withdrawing pyrazyl group or triazyl group, respectively. Therefore, it may be inferred that the bathochromic shifts of *λ*^abs-solid^_max_ and λ^fl-solid^_max_ observed in OUJ-2 by changing from the crystalline state to the amorphous state were caused due to the strong dipole–dipole interactions, whereas the non-obvious b-MFC for OUY-2 is ascribable to small changes in the dipole–dipole interactions between the crystalline and amorphous states due to its small dipole moment (*μ*_g_ = 1.4 debye), as with the case of non-obvious MFC for D–π–A-type fluorescent dyes with small dipole moments (*μ*_g_ = *ca.* 1–2 debye) in our previous study.^15^ On the other hand, it is speculated that the moderate ICT characteristics of OUK-2 may induces the h-MFC due to the twisting and distortion between the D–π moiety and A unit by grinding, as well as apparent b-MFC due to the strong dipole–dipole interactions which was suggested by the bathochromic shift of the shoulder fluorescence band by grinding. Meanwhile, the *Φ*_fl_ values in the amorphous state were slightly greater than those in the crystalline state, suggesting that the short-range π–π interactions between the dye molecules in the amorphous state discouraged the delocalization of excitons unlike the case of the long-range intermolecular π–π interactions between the dye molecules in the crystalline state. Consequently, this work reveals that (D–π–)_2_A fluorescent dyes possessing dipole moments of *ca.* 3 debye as well as ICT characteristics make it possible to activate the MFC. Moreover, it was found that a (D–π–)_2_A structure possessing intense ICT characteristics is necessary to exhibit pronounced MFC, judging from the fact that the (D–π–)_2_Ph fluorescent dye OTK-2 without any electron-withdrawing moiety but with a relatively strong dipole moment (*μ*_g_ = 4.0 debye) showed the non-obvious MFC. However, further fundamental studies to gain insight into the correlation between the ICT characteristics as well as the dipole moment and the MFC characteristics of (D–π–)_2_A fluorescent dyes, that is, an investigation into MFC of (D–π–)_2_A fluorescent dyes with dipole moments over 3 debye, are absolutely necessary and will be reported in a subsequent paper.

## Conclusions

We have found that (D–π–)_2_A-type azine-based fluorescent dyes possessing moderate or intense intramolecular charge-transfer (ICT) characteristics exhibit hypsochromic shift-type or bathochromic shift-type mechanofluorochromism (h-MFC or b-MFC): grinding of the recrystallized dyes induces hypsochromic or bathochromic shifts of the fluorescence maximum wavelengths. The experimental results and molecular orbital (MO) calculations demonstrated that the MFC of the (D–π–)_2_A-type azine-based fluorescent dyes was attributed to reversible switching between the crystalline state and amorphous state with changes in the intermolecular dipole–dipole and π–π interactions by grinding. We revealed that a (D–π–)_2_A structure possessing moderate or intense ICT characteristics is necessary to exhibit pronounced MFC: (D–π–)_2_A fluorescent dyes possessing dipole moments of *ca.* 3 debye as well as moderate or intense ICT characteristics make it possible to activate the MFC. Consequently, this work provided not only the correlation between the ICT characteristics as well as the dipole moment and MFC characteristics of (D–π–)_2_A fluorescent dyes but also a direction in the molecular design toward creating mechanofluorochromic dyes based on the dipole moment.

## Experimental

### General

Melting points were measured with an AS ONE ATM-02. IR spectra were recorded on a SHIMADZU IRTracer-100 by ATR method. ^1^H NMR and ^13^C NMR spectra were recorded on a Varian-500 FT NMR spectrometer. High-resolution mass spectral data by APCI were acquired on a Thermo Fisher Scientific LTQ Orbitrap XL. Photoabsorption spectra of solutions were observed with a Shimadzu UV-3600 plus spectrophotometer. Photoabsorption spectra of solids were recorded by a Shimadzu UV-3600 plus spectrophotometer with a calibrated integrating sphere system. Fluorescence spectra of solutions and solids were measured with a HORIBA FluoroMax-4 spectrofluorometer. Fluorescence quantum yields in solution and in the solid state were determined using a HORIBA FluoroMax-4 spectrofluorometer with a calibrated integrating sphere system. Fluorescence decay measurements were performed on a HORIBA DeltaFlex modular fluorescence lifetime system using a Nano LED pulsed diode excitation source (451 nm). Powder X-ray diffraction measurements were performed on a Rigaku MiniFlex600-C/CM diffractometer with Cu Kα radiator. Differential scanning calorimetry was carried out using a Shimadzu DSC-60. Load measurements were performed with an IMADA push–pull scale & digital force gauge (ZP-200/V). Densities of solids were evaluated with a Shimadzu AUW220D electronic balance equipped with an SMK-401 saucer by the Archimedian method. Semi-empirical calculations were carried out with the WinMOPAC Ver. 3.9 package (Fujitsu, Chiba, Japan), where geometry calculations of the compounds in the ground state were made using the AM1 method. Dipole moments and HOMO and LUMO energy levels of the compounds were also evaluated from INDO/S calculations.

## Conflicts of interest

There are no conflicts to declare.

## Supplementary Material

RA-012-D2RA02431D-s001
