# Supplementary material for: Mechanofluorochromism of (D–π–)2A-type azine-based fluorescent dyes
Source: RSC Adv. 2022 May 10;12(22):13797–809. doi: 10.1039/d2ra02431d (PMC9089242; doi:10.1039/d2ra02431d)
Supplement: RA-012-D2RA02431D-s001 [file RA-012-D2RA02431D-s001.pdf]

Electronic Supplementary Material (ESI) for RSC Advances.
This journal is © The Royal Society of Chemistry 2022

Supplementary Information

Mechanofluorochromism of (D–π–)$_2$A-type azine-based fluorescent dyes

Kosuke Takemura, Keiichi Imato and Yousuke Ooyama*

Applied Chemistry Program, Graduate School of Advanced Science and Engineering, Hiroshima University, 1-4-1 Kagamiyama, Higashi-Hiroshima 739-8527, Japan. E-mail: yooyama@hiroshima-u.ac.jp

Synthesis of 7,7'-(1,3-phenylenebis(thiophene-5,2-diyl))bis(9-butyl-*N*,*N*-diphenyl-9*H*-carbazol-2-amine) OTK-2. A solution of **1** (1.00 g, 1.57 mmol), 1,3-diiodobenzene (0.156 g, 0.47 mmol), and $Pd(PPh_3)_4$ (0.018 g, 0.016 mmol) in toluene (10 mL) was stirred for 32 h at 110 ºC under an argon atmosphere. The reaction mixture was diluted with water, and then, the solution was extracted with dichloromethane. The dichloromethane extract was dried over anhydrous $MgSO_4$, filtrated, and concentrated. The residue was chromatographed on silica gel (ethyl acetate : hexane = 1 : 4 and then, dichloromethane : hexane = 1 : 4 as eluent) to give **OTK-2** (0.463 g, yield 58%) as a light yellow solid; m.p. 249–251 °C; FT-IR (ATR): $\tilde{\nu}$ = 1589, 1490, 1456 cm^{-1}; ^1H NMR (500 MHz, CD_2Cl_2): δ = 0.89 (t, J = 7.3 Hz, 6H), 1.28–1.36 (m, 4H), 1.75–1.82 (m, 4H), 4.21 (t, J = 7.0 Hz, 4H), 6.95 (dd, J = 1.8 and 8.4 Hz, 2H), 7.01–7.04 (m, 4H), 7.13–7.16 (m, 10H), 7.25–7.29 (m, 8H), 7.45–7.49 (m, 5H), 7.55 (d, J = 8.0 Hz, 2H), 7.60–7.64 (m, 4H), 7.94 (d, J = 8.4 Hz, 2H), 7.97 (s, 1H), 8.01 (d, J = 8.1 Hz, 2H) ppm; ^{13}C NMR (125 MHz, CD_2Cl_2): δ = 14.05, 20.87, 31.46, 43.05, 105.04, 105.95, 117.54, 117.62, 118.76, 120.46, 121.18, 122.80, 122.95, 122.99, 124.24, 124.36, 124.90, 124.99, 129.56, 130.00, 131.36, 135.46, 141.69, 142.74, 142.85, 145.74, 146.92, 148.63 ppm; HRMS (APCI): m/z (%): $[M+H^+]$ calcd. for $C_{70}H_{59}N_4S_2$, 1019.41811; found 1019.41772.

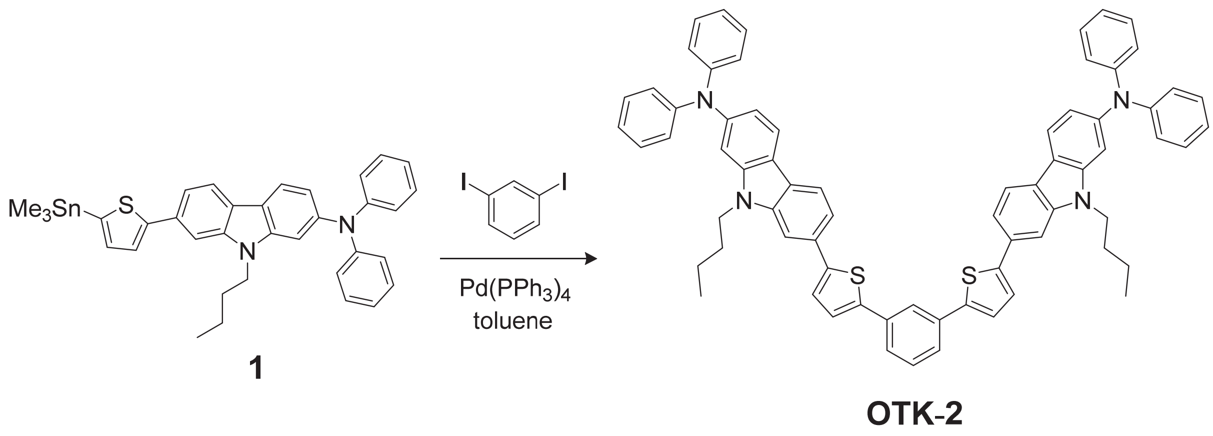

Scheme S1 Synthesis of **OTK-2**.

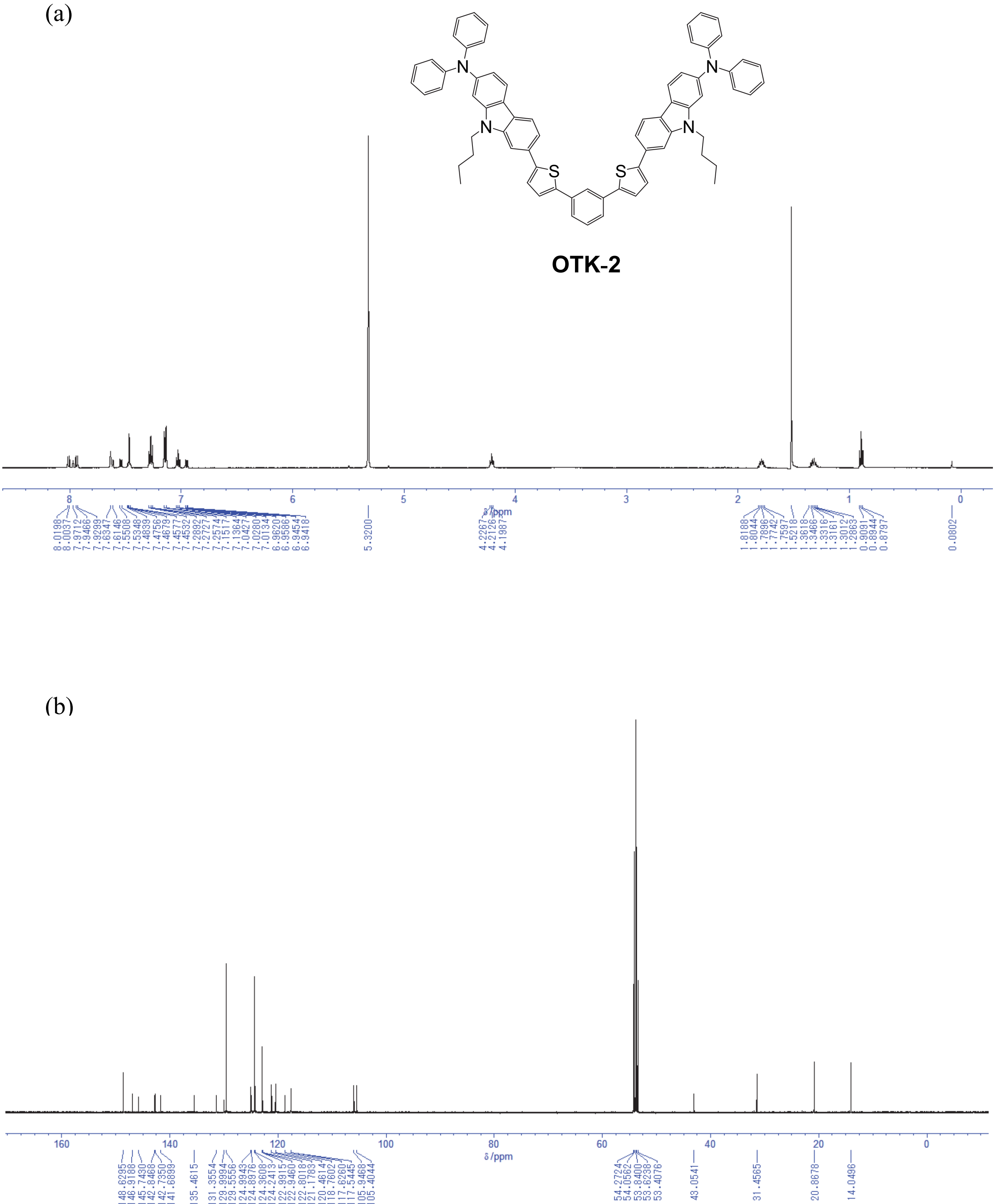

Fig. S1 (a) ^{1}H NMR (500 MHz) and (b) ^{13}C NMR (125 MHz) spectra of **OTK-2** in CD_2Cl_2.

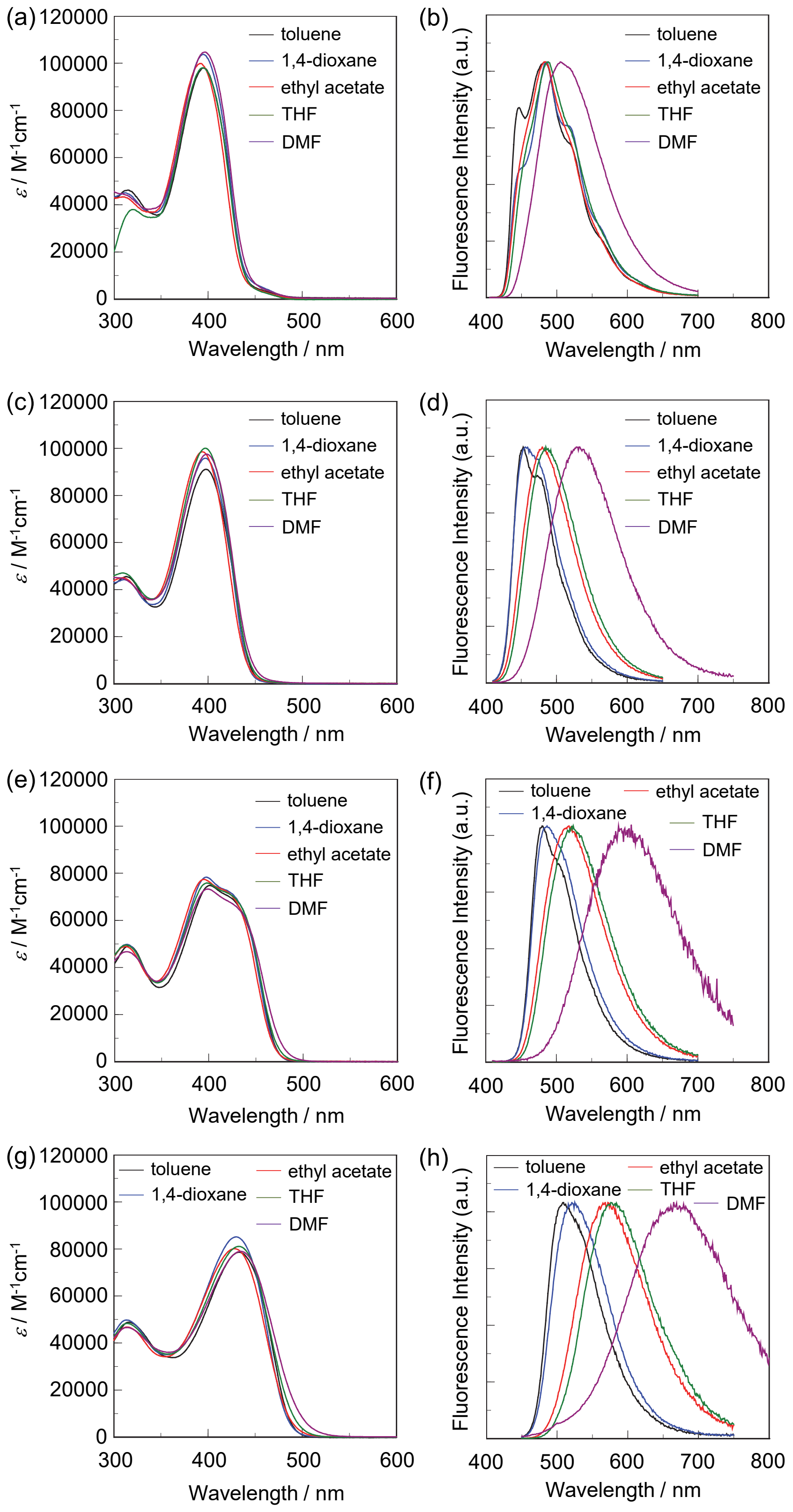

Fig. S2 (a) Photoabsorption and (b) fluorescence (λ^{ex} = ca. 400 nm) spectra of **OTK-2** in various solvents. (c) Photoabsorption and (d) fluorescence (λ^{ex} = ca. 400 nm) spectra of **OUY-2** in various solvents. (e) Photoabsorption and (f) fluorescence (λ^{ex} = ca. 400 nm) spectra of **OUK-2** in various solvents. (g) Photoabsorption and (h) fluorescence (λ^{ex} = ca. 430 nm) spectra of **OUJ-2** in various solvents.

Lippert–Mataga equation [eqn (1)]:

$$\nu_{st} = \frac{1}{4\pi\varepsilon_0} \cdot \frac{2\Delta\mu^2}{hca^3}\Delta f + \text{Const.} \qquad (1)$$

where

$$\Delta f = \frac{\varepsilon - 1}{2\varepsilon + 1} - \frac{n^2 - 1}{2n^2 + 1} \qquad (2)$$

In the above equations, ν_{st} is the Stokes shift, ε_0 is the vacuum permittivity, h is Planck's constant, c is the velocity of light, a is the Onsager radius of a dye molecule (7.74 Å, 7.81 Å, 7.99 Å, and 7.91 Å for **OTK-2**, **OUY-2**, **OUK-2**, and **OUJ-2**, respectively, estimated from DFT calculations at the B3LYP/6-31G(d,p) level), $\Delta\mu = \mu_e - \mu_g$ is the difference in the dipole moment of a dye between the excited (μ_e) and ground (μ_g) states, ε and n are the static dielectronic constant and refractive index of the solvent, respectively, and Δf is the orientation polarizability. On the basis of eqns (1) and (2), $\Delta\mu$ can easily be evaluated from the slope of a plot of ν_{st} against Δf (Fig. 3d; the Lippert–Mataga plot).

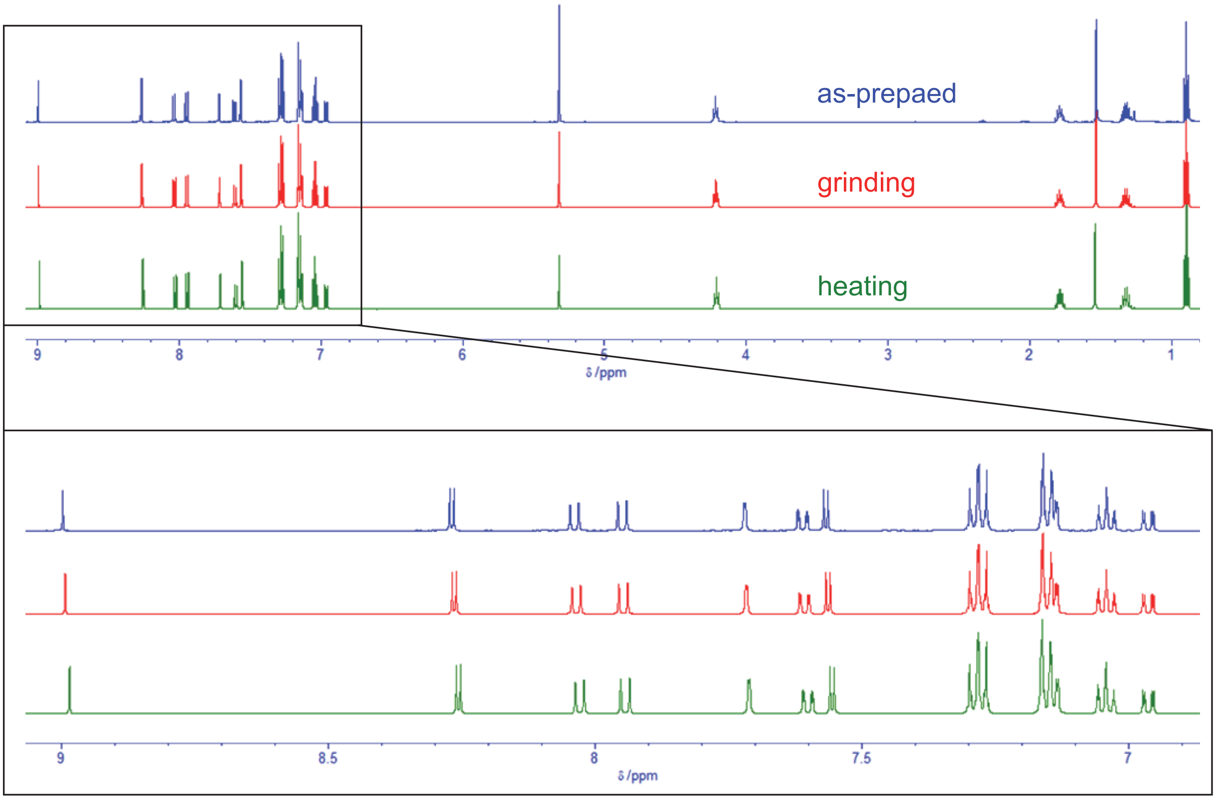

Fig. S3 ^{1}H NMR (500 MHz) spectra of as-prepared microcrystals, the ground solids, and the heated solids for **OUJ-2** in CD_2Cl_2.

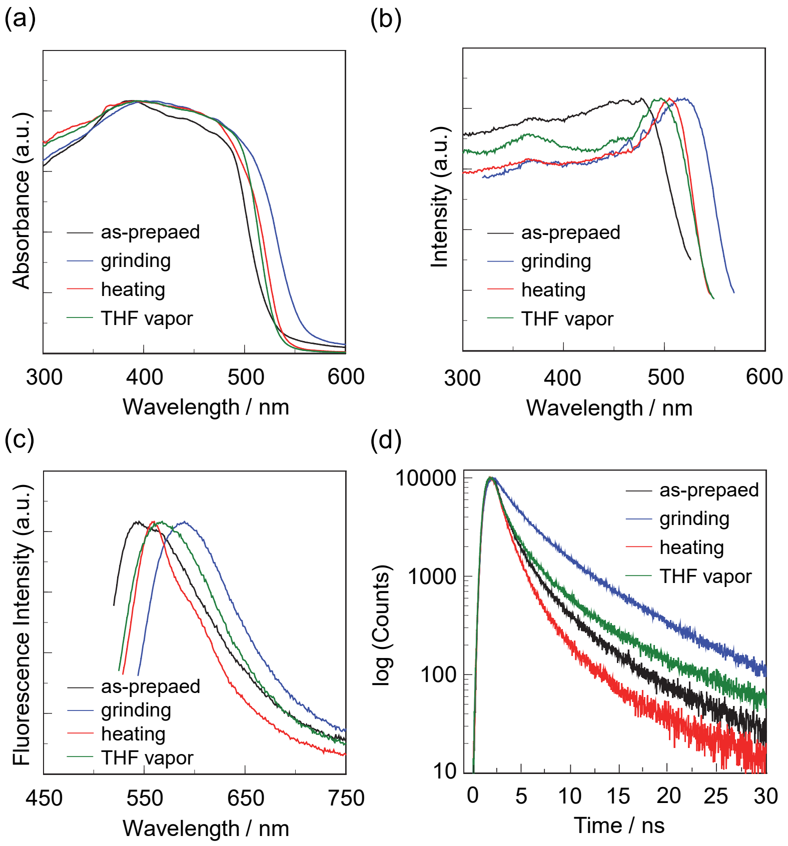

Fig. S4 (a) Solid-state UV-Vis diffuse reflection–absorption, (b) fluorescence excitation, and (c) fluorescence spectra ($\lambda^{ex} = \lambda_{max}^{ex\text{-}solid}$), and (d) fluorescence decay profiles of **OUJ-2** before and after grinding, after heating the ground solid at 200 ºC, and after exposure of the ground solid to THF vapor at 25 ºC for 15 min.

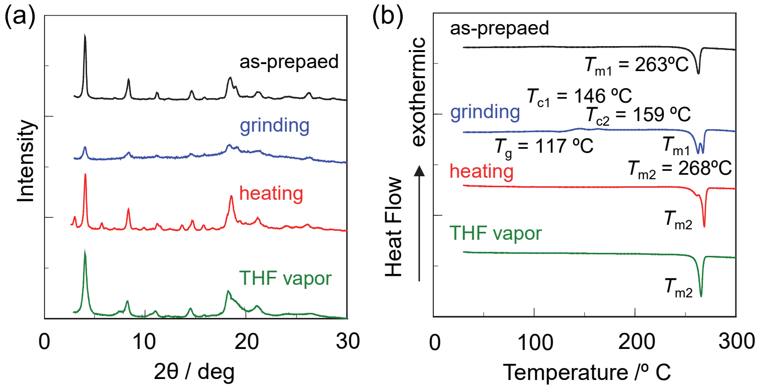

Fig. S5 (a) XRD patterns and (b) DSC curves (heating process from 25 ºC to 300 ºC with a scan rate of 10 ºC min^{-1}) of **OUJ-2** before and after grinding, after heating the ground solid at 200 ºC, and after exposure of the ground solid to THF vapor at 25 ºC for 15 min.

Table S1 Optical data of **OTK-2**, **OUY-2**, **OUK-2** and **OUJ-2** in various solvents

Dye	Solvent	λ_{max}^{abs} /nm (ε_{max}/M^{-1} cm^{-1})	λ_{max}^{fl}/nm (Φ_{fl})^{a}	SS^{b}/cm^{-1}	τ_{fl}/ns^{c}
**OTK-2**	Toluene	395 (98000)	447 (0.36)	2945	0.62
	1,4-Dioxane	395 (103800)	449 (0.36)	3045	0.68
	Ethyl acetate	391 (100000)	482 (0.36)	4829	0.60
	THF	395 (98100)	488 (0.42)	4825	0.69
	DMF	397 (105000)	505 (0.59)	5387	1.07
**OUY-2**	Toluene	398 (91100)	453 (0.38)	3051	0.82
	1,4-Dioxane	398 (95800)	455 (0.40)	3147	1.00
	Ethyl acetate	394 (98500)	480 (0.39)	4547	1.17
	THF	397 (100000)	485 (0.58)	4570	1.29
	DMF	399 (97500)	533 (0.59)	6300	2.33
**OUK-2**	Toluene	401 (74800)	480 (0.48)	4104	1.60
	1,4-Dioxane	397 (78300)	487 (0.62)	4655	1.91
	Ethyl acetate	398 (75800)	518 (0.55)	5820	2.14
	THF	394 (77400)	524 (0.65)	6296	1.95
	DMF	399 (73300)	588 (0.14)	8055	1.71
**OUJ-2**	Toluene	433 (78500)	509 (0.81)	3448	1.92
	1,4-Dioxane	430 (85100)	525 (0.86)	4208	2.20
	Ethyl acetate	428 (80100)	568 (0.72)	5758	2.78
	THF	433 (81100)	576 (0.72)	5733	2.93
	DMF	435 (78900)	665 (0.09)	7950	–[d]

[a] Fluorescence quantum yields (Φ_{fl}) were determined by using a calibrated integrating sphere system ($\lambda^{ex} = \lambda_{max}^{abs}$). [b]Stokes shift. [c] Fluorescence lifetime. [d] Due to feeble fluorescence properties.
